# STIM1 functions as a proton sensor to coordinate cytosolic pH with store-operated calcium entry

**DOI:** 10.1016/j.jbc.2024.107924

**Published:** 2024-10-23

**Authors:** Yilan Chen, Panpan Liu, Ziyi Zhong, Hanhan Zhang, Aomin Sun, Youjun Wang

**Affiliations:** 1 Beijing Key Laboratory of Gene Resource and Molecular Development, College of Life Sciences, Beijing Normal University, Beijing, China; 2 Key Laboratory of Cell Proliferation and Regulation Biology, Ministry of Education, College of Life Sciences, Beijing Normal University, Beijing, China

**Keywords:** STIM1, intracellular pH (pH_i_), cardiac hypertrophy, SOCE, calcium signaling, FRET imaging

## Abstract

The meticulous regulation of intracellular pH (pH_i_) is crucial for maintaining cellular function and homeostasis, impacting physiological processes such as heart rhythm, cell migration, proliferation, and differentiation. Dysregulation of pH_i_ is implicated in various pathologies such as arrhythmias, cancer, and neurodegenerative diseases. Here, we explore the role of STIM1, an ER calcium (Ca^2+^) sensor mediating Store Operated Ca^2+^ Entry (SOCE), in sensing pH_i_ changes. Our study reveals that STIM1 functions as a sensor for pH_i_ changes, independent of its Ca^2+^-binding state. Through comprehensive experimental approaches including confocal microscopy, FRET-based sensors, and mutagenesis, we demonstrate that changes in pH_i_ induce conformational alterations in STIM1, thereby modifying its subcellular localization and activity. We identify two conserved histidines within STIM1 essential for sensing pH_i_ shifts. Moreover, intracellular alkalization induced by agents such as Angiotensin II or NH_4_Cl enhances STIM1-mediated SOCE, promoting cardiac hypertrophy. These findings reveal a novel facet of STIM1 as a multi-modal stress sensor that coordinates cellular responses to both Ca^2+^ and pH fluctuations. This dual functionality underscores its potential as a therapeutic target for diseases associated with pH and Ca^2+^ dysregulation.

The regulation of intracellular protons (H^+^) and calcium ions (Ca^2+^) within cells is pivotal for coordinating fundamental physiological and cellular processes such as maintaining heart rhythm (1), regulating cell migration (2), proliferation, and differentiation (3). Aberrant intracellular pH (pH_i_) and Ca^2+^ signals are associated with various pathological conditions (4), including arrhythmias (5), oncogenesis (6, 7), and the progress of neurodegenerative diseases (8). This highlights the delicate balance these ions maintain between physiological integrity and pathological disruption. Amidst this complex interplay of cellular processes, the relationship between pH_i_ and Ca^2+^ signals emerges as a focal point of cellular signaling research (3, 9).

The endoplasmic reticulum (ER) Ca^2+^ Store Operated Ca^2+^ Entry (SOCE) is a ubiquitous Ca^2+^ influx pathway in animal cells (10) Prototypical SOCE is mediated by Ca^2+^ release-activated Ca^2+^ (CRAC) channel composed of STIM1, the ER Ca^2+^ sensor, and Orai1, the channel pore-forming protein on the plasma membrane (PM) (11). STIM1 contains a Ca^2+^-binding EF-hand, and sterile alpha motif (EF-SAM) in the ER lumen and a CRAC activation domain (CAD) (12) or STIM-Orai activating region (SOAR) (13) in the cytosol. At rest, the SOAR domain is masked by an “auto-inhibitory clamp” formed by the cytoplasmic coiled-coil 1 (CC1) region. In response to the depletion of ER Ca^2+^ stores, STIM1 EF-SAM domain releases Ca^2+^ and dimerizes, triggering a conformational change in CC1 that unleashes SOAR (14, 15, 16). Activated STIM1 molecules oligomerize and become enriched in the ER-PM junctions to form puncta. The exposed STIM1 SOAR motif then couples with and activates Orai1, leading to the formation of CRAC channels and triggering SOCE (11).

Beyond its role in Ca^2+^ sensing, STIM1 has been found to respond to various types of cellular stress signals (17, 18), such as temperature (19, 20, 21), reactive oxygen species (22), and hypoxia (18, 23). This multifaceted responsiveness suggests that STIM1 may play a broader role in coordinating cellular responses to diverse physiological and pathological conditions. Additionally, the sensitivity of the SOCE process to pH (24, 25) implies the intriguing possibility that STIM1 could directly perceive fluctuations in pH_i_, although this remains to be conclusively demonstrated.

Here, we have developed pH-insensitive fluorescent tools to monitor STIM1 activation and examined the impact of pH_i_ changes on STIM1 function. Our findings reveal that, beyond its roles as an ER Ca^2+^ sensor, STIM1 can sense fluctuations in cytosolic pH_i_ through two conserved histidine residues. The release of H^+^ from these residues activates STIM1, thereby enhancing STIM1-mediated SOCE and promoting cardiac hypertrophy. This capability of STIM1 to integrate signals from both Ca^2+^ and pH_i_ alterations underscores its versatility as a molecular switch, crucial for modulating cellular responses to ensure survival and functionality across diverse environmental and metabolic conditions.

## Results and discussion

### STIM1 responds to intracellular pH changes with minimal dependence on its Ca^2+^ binding status

**Figure 1:**
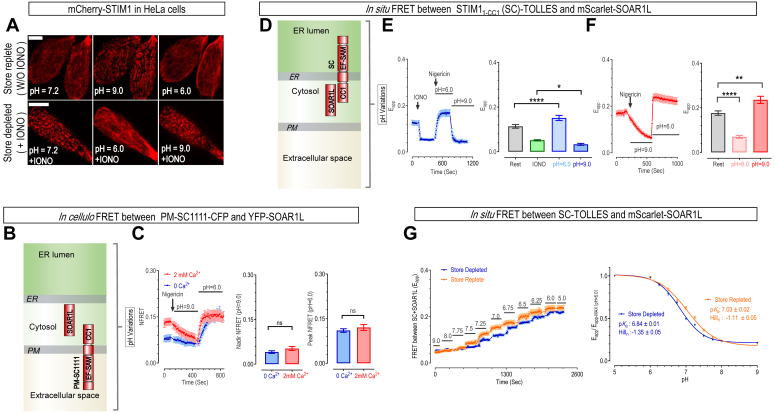
**STIM1 dose-dependently responds to changes in intracellular pH (pH**_**i**_**), with minimal dependence on adjacent Ca**^**2+**^**levels in HeLa cells.** Nigericin (10 μM), a H^+^/K^+^ ionophore, was used with high K^+^ buffer solutions at different extracellular pH (pH_e_) to adjust pH_i_. This allows pH_i_ to be controlled by matching it with pH_e_. *A*, typical confocal imaging results showing effects of pH_i_ changes on subcellular distribution of mCherry-STIM1 transiently expressed in HeLa cells at basal or ER Ca^2+^ depleted conditions. *Top*: cells under basal, or ER Ca^2+^ store replete conditions. *Bottom*: cells with ER Ca^2+^ store depleted by 5-min bath using 2.5 μM ionomycin (IONO), a Ca^2+^ ionophore (Scale bar: 10 μm, n = 3). *B* and *C*, *in cellulo* responses of PM-localized STIM1 to concurrent alterations in pH_e_ and pH_i_. Schematic representation of the subcellular distribution of the two STIM1 constructs tested for FRET imaging: YFP-STIM1_343-491_(SOAR1L) and engineered PM-localized STIM1_1-CC1_ (PM-SC1111)-CFP. Cartoon illustration of the subcellular distribution of these two constructs within cells (Refer to Fig. S1 for detailed description of these constructs). *B*, *left*: representative curves of FRET responses between YFP-SOAR1L and PM-SC1111–CFP monitored in nominally 0Ca^2+^ and 2 mM Ca^2+^ solution. *Middle* and *left*, statistics (*C*) (ns, *p* > 0.05, unpaired Student's *t* test, error bars denote SEM, n = 3). *D–F*, *in situ* responses of STIM1 to pH_i_ changes. Cartoon representation of acid-resistant FRET tools employed for monitoring the activation status of STIM1: mScarlet-SOAR1L and STIM1_1-CC1_ (SC)-Tolerance of Lysosomal Environments (TOLLES). TOLLES is an acid-resistant CFP variant (*D*). FRET responses between SC-TOLLES and mScarlet-SOAR1L in HeLa STIM1-STIM2 double knock-out (SK) cells. The measurements were conducted before altering pH_i_, with two conditions represented: with IONO treatment (*E*) *left*, representative curve; *right*, statistics (two-way ANOVA, F (3, 132) = 126.7, *p* < 0.0001;) (Rest *versus* pH = 6.0, *p* < 0.0001; IONO *versus* pH = 9.0, *p* = 0.049).and without IONO treatment (one-way ANOVA, F (2, 165) = 53.35, *p* < 0.0001) (pH = 6.0, *p* < 0.0001; pH = 9.0, *p* = 0.001). *F*, *left*, representative curve; *right*, statistics (error bars denote SEM, n = 3). *G*, *in situ* H^+^ titrations of FRET responses between transiently co-expressed SC-TOLLES and mScarlet-SOAR1L in HeLa SK cells. *Left*: typical traces; *right*: dose-response curves (n = 3).

To investigate whether STIM1 *per se* is sensitive to intracellular pH (pH_i_) changes, we overexpressed STIM1 tagged with mCherry, a more acid-resistant fluorescent protein (26) in HeLa cells, and assessed the impact of pH changes on its subcellular distribution. We manipulated the pH_i_, which includes both cytoplasmic and endoplasmic reticulum (ER) luminal pH (Fig. S2*A*), using nigericin (10 μM)-containing bath solution with varying pH. Nigericin is an H^+^/K^+^ ionophore that allows the equilibration of pH_i_ with extracellular pH (pH_e_), which means pH_i_ equals pH_e_ (27). In response to intracellular alkalization (pH_i_ = 9.0), STIM1 changed from a uniform ER-like distribution to an aggregated puncta even under basal conditions (Fig. 1*A*, top left and middle). Furthermore, when the pH_i_ was changed to 6.0, the previously formed puncta reverted to an ER-like distribution (Fig. 1*A*, top right). Next, we depleted the ER Ca^2+^ stored with a 5-min bath using 2.5 μM ionomycin (IONO), a Ca^2+^ ionophore. STIM1 puncta in store-depleted cells disappeared as pH_i_ decreased from physiological conditions to acidic level (pH_i_ = 6.0) (Fig. 1*A*, bottom right and middle). Upon increasing the pH_i_ to 9.0, STIM1 puncta re-formed. These results indicate that pH_i_ reversibly influences the subcellular localization of STIM1 independent of the store status, suggesting that STIM1 may possess the capability to sense pH_i_ independently of sensing Ca^2+^.

To validate directly that STIM1 can detect pH_i_ changes independent of its Ca^2+^-binding, we employed a FRET-based tool to monitor STIM1 *per se* activation in live cells (28). Specifically, this tool consists of two fluorescently labeled fragments of STIM1, STIM1_1-CC1_ (SC), and SOAR1L (Fig. S1*A*). Under resting conditions, SOAR1L is docked to the CC1 domain of the SC construct, resulting in higher FRET efficiency between the two components. Upon activation, they dissociate, leading to a decreased FRET signal (Fig. S1*B*). This tool enables real-time, dynamic monitoring of STIM1 activation at the nanoscale in live cells (28). Both our group and others have successfully employed this method to dissect the activation mechanism of STIM1 and STIM2 (14, 15, 29, 30, 31). Furthermore, to precisely control the degree of STIM1 activation with specific Ca^2+^ concentrations, we employed a Plasma Membrane-localized STIM1_1-CC1_ (PM-SC) tool (construction strategy shown in Fig. S1*C*) to measure STIM1 activation *in cellulo*. We have previously demonstrated that PM-SC constructs effectively localize to the PM and can accurately sense fluctuations in extracellular Ca^2+^ levels (31) (Fig. S1*C*). Featuring a Ca^2+^-sensing EF-SAM domain positioned at the extracellular side of PM, this tool allows precise control of its Ca^2+^-binding status by manipulating extracellular bath solutions with different Ca^2+^ concentrations. To minimize potential biases arising from pH-dependent changes in YFP & CFP fluorescence (18), we utilized NFRET (32), which represents FRET signals normalized against CFP and YFP fluorescence. We then compared the NFRET responses of PM-SC and SOAR1L to pH changes: under mostly Ca^2+^-bound basal conditions (2 mM Ca^2+^) or activated apo-state (nominally Ca^2+^ free) (31) (Fig. 1*B*).

In an imaging solution containing 2 mM Ca^2+^, the majority of PM-SC1111 molecules, a STIM1 version of PM-SC, bind to Ca^2+^ and position SOAR1L near PM, resulting in an elevated basal NFRET signal with SOAR1L. Surprisingly, by simply switching the bath solution to an alkaline one (pH 9.0) without changing extracellular Ca^2+^ levels, NFRET decreased to a level comparable to that in nominally Ca^2+^ free solution with physiological pH (7.2), or under STIM1-activated condition (Fig. 1*C*). Conversely, the low resting NFRET signal in a nominally Ca^2+^-free solution, mediated by the activated apo-STIM1, was reversed back to high levels seen at physiological pH with 2 mM Ca^2+^, or under STIM1-rest conditions (Fig. 1*C*). This result demonstrates that STIM1 can respond to pH changes under Ca^2+^-bound basal conditions or activated apo-state. Of note, the plateau values of the NFRET signal between PM-SC and SOAR1L in alkaline conditions (pH 9.0) remained largely unchanged despite variations in Ca^2+^ concentrations (Fig. 1*C*, middle panel). Similarly, those observed under acidic circumstances (pH 6.0) showed no significant difference across both Ca^2+^ conditions (Fig. 1*C*, right panel). Collectively, these results demonstrate that pH may act independently of Ca^2+^, directly inducing conformational changes in STIM1 and thereby altering its activation status.

To more accurately evaluate pH-induced responses of STIM1, we replaced the original CFP & YFP proteins with a more acid-resistant fluorescent protein pair: mScarlet and a CFP variant named “Tolerance of Lysosomal Environments” (TOLLES) (33). The fluorescence of these two proteins is indeed insensitive to pH variations tested (Fig. S2*C*). Unfortunately, PM-SC-TOLLES did not exhibit satisfactory PM localization, preventing further in-cell characterization. Consequently, we employed the original CFP-YFP pair along with the less pH-sensitive NFRET parameter to assess the activities of the PM-SC constructs. While SC-TOLLES showed correct ER-like distribution (Fig. S2*D*). Following store depletion with IONO, the FRET signals rapidly decreased (Fig. 1*E*), demonstrating its efficacy in reporting STIM1's conformational changes upon activation. We thus utilized the more precise, system-independent apparent FRET efficiency (E_app_) for all SC measurements.

We next examined the FRET responses between SC-TOLLES and mScarlet-SOAR1L to pH_i_ changes *in situ*, under both store-replete and -depleted conditions (Fig. 1*D*). Consistent with the *in cellulo* results, under store-depleted conditions, as pH_i_ decreased from physiological to acidic (pH 6.0), FRET signals increased from low to high levels, reflecting the transition of STIM1 from an activated state back to a resting state. Subsequently, an increase in pH_i_ to 9.0 resulted in a reduction of FRET signals, indicating its return to an activated state (Fig. 1*E*). Similarly, with replete stores, intracellular alkalization lowered the FRET signals while subsequent acidification increased the FRET signals to a plateau that was slightly higher than basal (Fig. 1*F*). These findings collectively demonstrate that pH can independently influence STIM1, inducing conformational changes and thereby altering its activation status in its native environment, regardless of ER Ca^2+^ levels.

Intriguingly, the plateau FRET signals under acidic conditions slightly surpass those observed under store-replete conditions at physiological pH_i_ (Fig. 1, *E* and *F*, bar chart), indicating that intracellular acidification heightened the basal auto-inhibition of STIM1. Conversely, intracellular alkalization further subtly reduced the FRET signals between SC and SOAR1L following store depletion at physiological pH_i_, indicating elevated activation under alkaline conditions. These results thus demonstrate that pH_i_ variations modulate the Ca^2+^-dependent activation status of STIM1.

Nevertheless, these findings collectively demonstrate STIM1 as pH_i_ sensors, both *in cellulo* and *in situ*.

To quantitatively characterize STIM1's responses to changes in pH_i_, we performed *in situ* [H^+^]-titration using our FRET tool, SC-TOLLES, and mScarlet-SOAR1L, under both store-replete and -depleted conditions in HeLa STIM1-STIM2 double knock-out (SK) cells (Fig. 1*G*). The results showed that FRET signals between SC and SOAR1L are dose dependently altered by H^+^ concentrations ([H^+^]), where lower H^+^ concentrations (higher pH level) were associated with diminished FRET signals, indicative of increased activation of STIM1 In other words, fully protonated STIM1 under low pH would remain mostly inactive, while apo-STIM1 or deprotonated STIM1 would be active. With replete ER stores, the [H^+^] response of STIM1 exhibited a p*K*a of 7.03 and a Hill coefficient of 1.11. Upon store depletion, the observed p*K*a shifted to 6.84, with the Hill coefficient adjusted to 1.32. These results thus revealed subtle yet significant effects of ER Ca^2+^ levels on the pH_i_-sensing behavior of STIM1. Upon store depletion, the lowered p*K*a value and increased Hill number suggest that STIM1 exhibits heightened responsiveness to acidic conditions with more cooperativity. Nevertheless, the p*K*a of STIM1 under both conditions is close to pH 7.2, implying that STIM1 can sense fluctuations in physiological pH_i_.

Collectively, these findings establish STIM1 as an authentic pH_i_ sensor for physiological pH_i_ fluctuations, with minimal influence from Ca^2+^.

### STIM1's cytosolic region is pivotal for its pH-sensing ability

We next set out to pinpoint the crucial domains within STIM1 that are critical for pH sensing. We first ruled out the transmembrane (TM) domain due to its limited exposure to pH fluctuations within the ER lumen or cytosol. Adopting a 'divide-and-conquer' strategy, we then focused our investigation on the ER-luminal domain, as well as its cytosolic CC1 and SOAR1L regions.

**Figure 2:**
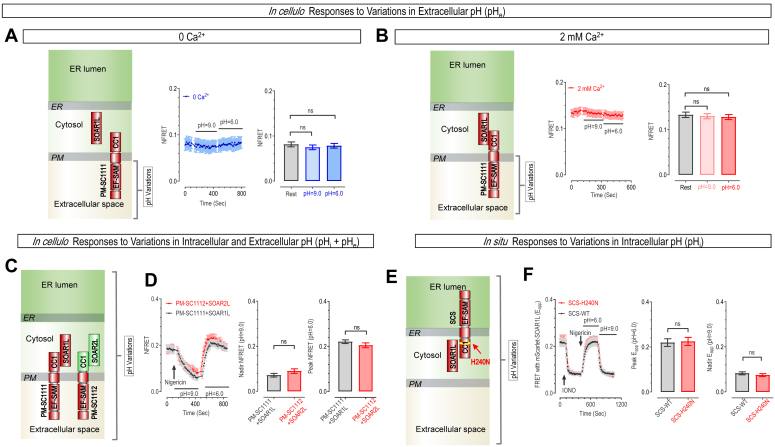
**Mapping critical regions essential for STIM1 in sensing changes in pH**_**i**_**within HeLa SK cells.***A* and *B*, perturbations in pH_e_ adjacent to EF-SAM region of PM-SC1111–CFP had no substantial impact on its FRET responses with YFP-SOAR1L. Recordings were carried out using cells immersed in nominally Ca^2+^ free solution (*A*) *left*: cartoon depiction of constructs used; Middle: representative curves; *right*: statistics (one-way ANOVA, F (2, 390) = 0.39, *p* = 0.67) (pH = 6.0, *p* = 0.65; pH = 9.0, *p* = 0.91), (error bars denote SEM, n = 3) or buffers containing 2 mM Ca^2+^ (*B*). *Left*: cartoon depiction; *middle*: representative curves; *right*: statistics (one-way ANOVA, F (2, 333) = 0.19, *p* = 0.83) (pH = 6.0, *p* = 0.92; pH = 9.0, *p* = 0.83), (error bars denote SEM, n = 3). *C* and *D*, under extracellular conditions with a high concentration of Ca^2+^ (30 mM), replacing the CC1-domain and SOAR1L with those of STIM2 (PM-SC1112-CFP and YFP-SOAR2L) had minimal impact on STIM1's response to changes in adjacent pH (Plasmid construction strategy: see Fig. S1). Schematic representation of STIM1/2 constructs tested (PM-SC1111-CFP + YFP-SOAR2L or PM-SC1112-CFP + YFP-SOAR1L). *C*, typical traces (*left*) and statistics (*middle and right*) (*D*) (ns, *p* > 0.05, unpaired Student's *t* test, error bars denote SEM, n = 3). *E* and *F*, STIM1-H240N mutation had no effect on *in situ* FRET responses between STIM1_1-261_(SCS)-TOLLES and mScarlet-SOAR1L. Schematic representation of STIM1 constructs tested (*E*). Representative traces (*left*) and statistics (*middle and right*) (*F*) (ns, *p* > 0.05, unpaired Student's *t* test, error bars denote SEM, n = 3).

We first assessed the involvements of the ER-luminal domain bearing the Ca^2+^-binding EF-SAM region in pH sensing, using the engineered PM-SC constructs with the original ER luminal domain now facing extracellular space (Fig. 2*A*, diagram on the left). We monitored the effect of changes in pH_e_ alone, or pH variations adjacent to the “original ER luminal region” of STIM1, on the FRET response of PM-SC1111 and SOAR1L. In a nominally Ca^2+^-free solution, the FRET signals between apo-PM-SC1111 and SOAR1L remained unchanged by pH_e_ changes (Fig. 2*A*). Similarly, in a bath solution containing 2 mM Ca^2+^, the FRET signals between the mostly Ca^2+^ bound PM-SC1111 and SOAR1L were insensitive to pH_e_ variations (Fig. 2*B*). Thus, regardless of Ca^2+^, alterations in pH around the N-terminal region did not significantly affect the FRET signals. These results thus demonstrate that the Ca^2+^-sensing ER luminal region of STIM1 does not respond to changes in pH.

We then moved on to examine the effects of STIM1 cytosolic regions, CC1 and SOAR1L, in pH sensing. We assessed the FRET response of PM-SC1111-CFP and YFP-SOAR1L to alterations of both pH_i_ and pH_e_. We used a bath solution that contained 30 mM Ca^2+^ to ensure full binding of STIM1 with Ca^2+^, or complete auto-inhibition of STIM1. In sharp contrast to results obtained with pH_e_ alterations alone (Fig. 2*B*), simultaneous intra- and extra-cellular alkalization resulted in a notable decrease in FRET signal, and a return to baseline levels upon subsequent acidification of STIM1 surrounding environments (Fig. 2*C*, black). Together, these two results clearly demonstrate the essential role of STIM1 cytoplasmic region in sensing pH.

### STIM1-H355N-H407N (2HN) showed unaltered Ca^2+^ sensing and minimal pH sensitivity

To map the critical amino acid residues responsible for pH sensing, we first examined the effects of swapping the STIM1 cytosolic fragments of our FRET tool with those of the STIM2 homolog. The results showed that the FRET responses of SOAR2L and chimeric PM-SC1112, or PM-SC1111 with its cytosolic CC1 replaced by STIM2-CC1 (Fig. S1*A*), overlapped with that of SOAR1L and PM-SC1111 (Fig. 2*C*, black *versus* red). Thus, the cytosolic region of STIM homologs showed the same pH sensitivity (Fig. 2*D*), indicating the pH-sensing residues are among those conserved ones in STIM1 and STIM2.

With a side-chain p*K*a value close to physiological pH, histidine residues play an essential role in many cellular pH sensors (34). Therefore, our attention is directed towards the conserved histidine residues within the cytosolic regions of both STIM1 and STIM2 (Fig. S3).

We first evaluated the effect of mutating H240, the sole conserved histidine within CC1, to the uncharged Asparagine (Asn, N) on pH sensing (Fig. 2*E*). A short SC fragment, STIM1_1-261_ (SCS), was used as it showed the same FRET responses with SOAR1L as SC to pH_i_-changes (Fig. S4*A*). The results showed that both the wild-type (WT) and H240N mutants responded similarly to pH fluctuations (Fig. 2*F*), revealing a minimal role of CC1 in pH sensing.

**Figure 3:**
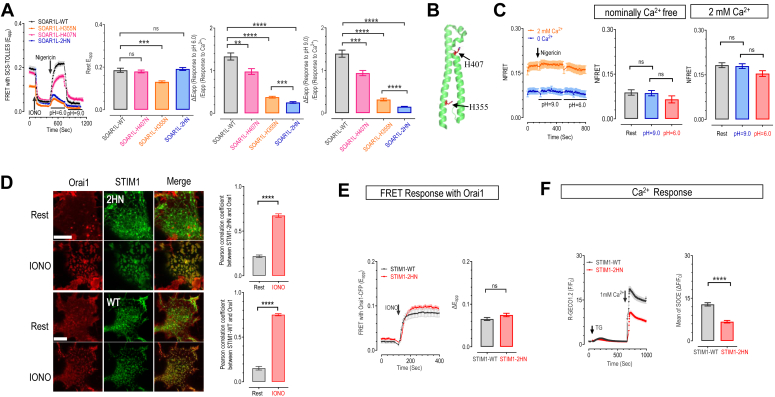
**Mutations at SOAR1L-H355-H407 minimally impact STIM1's coupling with Orai1 but significantly diminish its pH-sensing ability.***A*, FRET responses between SCS-TOLLES and mScarlet-SOAR1L WT (*black*), or its H407N (*pink*), H355N (*orange*), H355N-H407N mutant (2HN) (*blue*) in HeLa SK cells. *Left most*: typical curves; bar charts on the *right*: statistics of FRET signals. *Middle left*, basal FRET; *middle right and rightmost*, changes in FRET signals induced by acidification or alkalization, normalized against those resulting from store depletion. (*Middle left*: one-way ANOVA, F (3, 238) = 9.88, *p* < 0.0001) (H355N, *p* = 0.006; H407N, *p* = 0.922; 2HN, *p* = 0.91), (middle right: one-way ANOVA, F (3, 242) = 80.36, ∗∗∗∗*p* < 0.0001; ∗∗∗*p* = 0.0001, ∗∗*p* = 0.0096), (*right*: one-way ANOVA, F (3, 238) = 110.2, ∗∗∗∗*p* < 0.0001; ∗∗∗*p* = 0.0003) (error bars denote SEM, n = 3). *B*, a diagram illustrating the location of H355 and H407 residue on SOAR1 structure. *C*, FRET responses mediated by PM-SC1111-CFP and YFP-SOAR1L-2HN in HeLa SK cells. *Left*, representative traces; *middle & right*, statistics of pH-induced changes in FRET signal (*middle*: one-way ANOVA, F (2, 102) = 1.615, *p* = 0.2) (rest *versus* pH = 9.0, *p* = 0.99; pH = 9.0 *versus* pH = 6.0, *p* = 0.31) (*left*: one-way ANOVA, F = 3.14 *p* = 0.0453, rest *versus* pH = 9.0, *p* = 0.9643; pH = 9.0 *versus* pH = 6.0, *p* = 0.1074) (error bars denote SEM, n = 3). *D–F*, effects of 2HN mutation on the coupling of STIM1 with Orai1 and corresponding Ca^2+^ responses examined in HEK SK cells stably expressing Orai1-CFP (SKO cells). *D*, co-localization between Orai1-CFP (*red*) and transiently expressed STIM1-YFP or STIM1-H355N-H407N (2HN)-YFP (*green*) with Orai1-CFP (*red*) in HEK SKO cells pre- and post-store depletion. Store depletion induced by a 5-min bath application of 2.5 μM IONO. *Left*, typical confocal images. (*Uppermost and lower middle row on the left*: Store repleted; *upper middle lowest row*: Store depleted). (Scale bar: 10 μm, n = 3). Bar chart on the right, statistics. (n = 3, ∗∗∗∗*p* < 0.0001, unpaired Student's *t* test, error bars represent SEM). *E*, FRET responses mediated by Orai1-CFP and YFP-STIM1 or YFP-STIM1-2HN. Store depletion was induced by 2.5 μM IONO (*C*). *Left*, representative traces; *right*, statistics of IONO-induced increases in FRET signal (ns, *p* > 0.05, unpaired Student's *t* test, error bars denote SEM, n = 3). *F*, Ca^2+^ responses induced by thapsigargin (TG, 1 μM) in SKO cells transiently co-expressing R-GECO1.2, a low affinity red GECI, and YFP-STIM1 or YFP-STIM1-2HN. TG is a blocker of the ER Ca^2+^ pump to induce depletion of ER Ca^2+^ store and subsequent SOCE. *Left*, typical traces; *right*, statistics of TG-induced SOCE (n = 3, ∗∗∗∗*p* < 0.0001, unpaired Student's *t* test, error bars represent SEM).

Next, we investigated the roles of the four conserved histidine residues in STIM1-SOAR1L (H355, H395, H398, and H407) in pH sensing with mutagenesis and FRET imaging. SOAR1L-H395N-H398N mutant showed no notable impact (Fig. S4*B*), while H355N and H407N mutations displayed significant differences in pH-induced FRET responses compared to SOAR1L-WT (Fig. 3, *A* and *B*). The H407N mutation moderately affected FRET signals, while the SOAR1L-H355N mutant showed a more pronounced impairment in pH responses (Fig. 3*A*).

To distinguish between pH sensing impairments and CC1-SOAR1L interaction defects, we also assessed the impacts of mutations on FRET signals between SCS and SOAR1L constructs, both basal levels and changes triggered by IONO-induced ER Ca^2+^ store depletion. These Ca^2+^-dependent FRET changes served as indicators for CC1-SOAR1L interaction. Consistent with recent findings on CAD-H355A mutant (29), the basal FRET of the SOAR1L-H355N with SCS and corresponding IONO-induced decreases in FRET were lower than WT SOAR1L (Fig. 3*A*, middle left). This suggests that H355N mutation impairs the base auto-inhibitory CC1-SOAR interactions and corresponding ER-store-dependent changes. Interestingly, the dual mutation of both sites, H355N-H407N (2HN), resulted in a full restoration of the resting and IONO-induced changes in FRET signals, indicating that the dynamic ER store-dependent CC1-SOAR interactions are unaffected (Fig. 3*A*, black *versus* blue). We also evaluated the effect of 2HN mutation on the Ca^2+^ sensing ability of full-length STIM1 with confocal imaging. Results showed that, under physiological pH conditions, the subcellular distribution of STIM1-2HN remains similar to that of WT, both before and after store depletion (Fig. S4*C*). Both FRET and confocal imaging results demonstrate the unaltered Ca^2+^-sensing ability of STIM1-2HN. On the contrary, the pH responses of SOAR1L-2HN with SCS were greatly compromised, clearly demonstrating a separation of Ca^2+^ and pH sensing.

To cancel out artifacts caused by variations in CC1-SOAR1L coupling and better evaluate the pH sensing ability of SOAR1L variants, we normalized their pH responses against those trigged by store depletion, comparing them among SOAR1L variants (Fig. 3*A*, bar charts middle right and right). Results demonstrate that both H407N and H355N mutations inhibited pH responses, with the latter exhibiting a stronger effect, while the 2HN mutant displayed markedly diminished pH responses.

We further examined the effects of 2HN mutation on STIM1’s Ca^2+^ and pH sensing with PM-localized PM-SC1111 bathed in 2 mM Ca^2+^ or nominally free solution. Regardless of its Ca^2+^ binding status, the FRET signals between PM-SC1111 are no longer affected by pH changes (Fig. 3*C*). These results thus prove that H355 and H407 residues within SOAR1L are essential for the pH-sensing ability of STIM1. Together, these results firmly establish that the two conserved residues, H355-H407, are not involved in Ca^2+^ sensing, but are crucial for the pH sensing ability of STIM1.

### The pH-sensing STIM1-2H residues are involved in STIM1’s functional coupling with Orai1 to mediate SOCE responses

After confirming that STIM1-2HN can effectively sense ER Ca^2+^ store depletion, we proceed to evaluate its ability to couple with Orai1. We first monitored its ability to interact with Orai1 with confocal imaging. Similar to STIM1-WT (Fig. 3*D*, lower middle and lowest row), STIM1-2HN adopted a uniform ER-like distribution, with minimal colocalization with Orai1 (Fig. 3*D*, uppermost panels). Upon IONO-induced store depletion, STIM1-2HN also formed a large discrete puncta and co-localized with Orai1 (Fig. 3*D*, upper middle panels). The 2HN mutation did not significantly alter the IONO-induced co-localization between Orai1 and STIM1 (Pearson correlation coefficient, 0.67 ± 0.02). We then examined the effects of 2HN mutation on the FRET signals between STIM1 and Orai1. The basal FRET signals between Orai1 and STIM1-2N, as well as the IONO-induced corresponding changes, were similar to those between Orai1 and WT STIM1 (Fig. 3*E*), indicating that the 2HN mutation did not significantly impair its binding with Orai1.

Subsequently, we conducted Ca^2+^ imaging to examine the functional consequences of these two mutations by monitoring SOCE responses in HEK-Orai1 cells transiently expressing STIM1 variants. Interestingly, cells with STIM1-2HN expression exhibited smaller SOCE responses compared to those expressing WT STIM1 (Fig. 3*F*), indicating a compromised functional coupling with Orai1. This observation aligned with a previous study positioning H355 within the STIM-Orai association pocket (35). Likely, the STIM1-mediated gating of Orai1 was slightly impaired by 2HN mutation. Nonetheless, our findings collectively demonstrate that STIM1-2HN remains capable of inducing SOCE in response to store depletion through its interaction with Orai1.

### The enhanced STIM1-mediated SOCE is required for cardiac hypertrophy induced by intracellular alkalization in HL-1 cells

SOCE was shown essential for cardiac hypertrophy induced by hyperactivity of Na^+^/H^+^ exchanger 1 (NHE1) (36, 37), which reportedly altered pH_i_ in podocytes and monocytes (38, 39). However, the role of STIM1-pH_i_-sensing and its downstream events in hypertrophy remains unexplored. We therefore employed an HL-1 cell model of myocardial hypertrophy and examined corresponding changes in pH_i_ and Ca^2+^ responses after NHE1 activation (40, 41).

**Figure 4:**
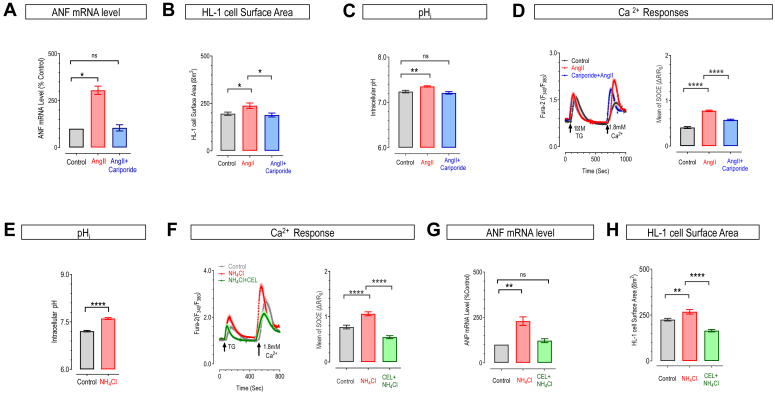
**Angiotensin II (Ang II) or NH**_**4**_**Cl may induce cardiac hypertrophy by enhancing STIM1-mediated SOCE through pH**_**i**_**alkalization in HL-1 Cells.***A–D*, Ang II-induced cardiac hypertrophy was accompanied with pH_i_ alkalization and enhancement of SOCE. Hypertrophy was induced by 24 h-incubation with 0.1 μM Ang II in serum-free medium. Control cells were compared with those treated with 0.1 μM Ang II alone, or with the combination of 1 μM cariporide, an inhibitor of Na^+^/H^+^ exchange inhibitor 1 (NHE1). Statistics of mRNA level of atrial natriuretic factor (ANF) (one-way ANOVA, F (2, 6) = 29.89, *p* = 0.0039) (Ctrl *versus* Ang II, *p* = 0.01; Ctrl *versus* AngII + cariporide, *p* = 0.986) (*A*) and cell surface area (*B*) mRNA levels were normalized to GAPDH (one-way ANOVA, F (2, 6) = 4.952, *p* = 0.0085) (Ctrl *versus* Ang II, *p* = 0.0231; Ang II *versus* AngII + cariporide, *p* = 0.260). Statistics of pH_i_, measured with a genetically encoded ratiometric pH sensor, pHmScarlet-mTurquoise2 (*C*). (One-way ANOVA, F (2, 166) = 9.283, *p* = 0.0002) (Ang II, *p* = 0.0016; AngII + cariporide, *p* = 0.6532). TG-induced Ca^2+^ responses showed by a cytosolic Ca^2+^ indicator, Fura-2. *Left*, typical traces; right, statistics of SOCE responses (*D*) (one-way ANOVA, F (2, 321) = 105.6, *p* < 0.0001) (Ctrl *versus* Ang II, *p* < 0.0001; Ang II *versus* AngII + cariporide, *p* < 0.0001). (n = 3, error bars denote SEM). *E*, statistics showing effects of 24 h-treatment with 40 mM NH_4_Cl on pH_i_. pH_i_ was measured using pHmScarlet-mTurquoise2. (n = 3, ∗∗∗∗*p* < 0.0001, unpaired Student's *t* test, error bars represent SEM). *F–H*, TG-induced Ca^2+^ responses or cardiac hypertrophic responses in controls, or cells following 24 h-treatment with 40 mM NH_4_Cl alone, or in combination with 1.5 μM celastrol (CEL), a specific inhibitor of SOCE. TG-induced Ca^2+^ signals *left*: representative curves; *right*: statistics of SOCE responses (one-way ANOVA, F (2, 389) = 43.78, *p* < 0.0001) (Ctrl *versus* NH_4_Cl, *p* < 0.0001; Ctrl *versus* NH_4_Cl + CEL, *p* < 0.0001) (*F*). Statistics of ANF mRNA level (one-way ANOVA, F (2, 8) = 17.58, *p* = 0.0012) (Ctrl *versus* NH_4_Cl, *p* < 0.0013; Ctrl *versus* NH_4_Cl + CEL, *p* = 0.5735) (*G*), cell surface area (one-way ANOVA, F (2, 282) = 33.04, *p* < 0.0001) (Ctrl *versus* NH_4_Cl, *p* = 0.002; Ctrl *versus* NH_4_Cl + CEL, *p* < 0.0001) (*H*). (n = 3, error bars denote SEM).

The hypertrophic response was induced by 24-h treatments with 0.1 μM angiotensin II (Ang II), a known activator of NHE1. The extent of cardiac hypertrophic response was indicated by the mRNA levels of atrial natriuretic factor (ANF) and changes in cell surface area (36, 37). After Ang II treatments, HL-1 cells showed elevated ANF mRNA levels and increased surface area compared to the control group, indicating successful hypertrophy induction (Red *versus* black bars, Fig. 4, *A* and *B*). Moreover, co-administration of Ang II with the NHE inhibitor cariporide (1 μM) completely nullified the induced effects, suggesting that the hypertrophic effects are directly linked to enhanced NHE function (Blue *versus* black bars, Fig. 4, *A* and *B*).

To quantitatively monitor basal pH_i_ levels, we tagged an acid-resistant CFP variant, mTurquoise2 (mTq2) (42), onto a recently developed genetically encoded pH indicator pHmScarlet (43) to generate the ratiometric pH sensor, pHmSc-mTq2 (Fig. 4*C*). It can minimize artifacts caused by variations in pHmScarlet expression levels without affecting the inherent pH-reporting properties of pHmScarlet (Fig. S2*B*). Results showed that after incubation with Ang II for 24 h, the basal pH_i_ level of HL-1 cells increased from 7.23 to 7.35. And this alkalization of cytosol was reversed by co-incubation with 1 μM cariporide (Fig. 4*C*). These results demonstrate that NHE1 activation can directly elevate the pH_i_ in HL-1 cells.

We next performed Ca^2+^ imaging to evaluate the impact of pH_i_-elevation on Ca^2+^ responses after Ang II treatments in HL-1 cells. Using the chemical Ca^2+^ indicator Fura-2, we monitored TG-induced Ca^2+^ release, as well as SOCE responses induced by subsequent Ca^2+^ re-addition. Results revealed no significant differences in TG-induced Ca^2+^ release among the three treatment groups, indicating no detectable changes in ER Ca^2+^ storage. However, we observed a significant increase in SOCE following Ang II treatment, with a notable decrease in SOCE upon co-treatment with cariporide, albeit not reaching the levels of the untreated group (Fig. 4*D*). These results collectively suggest that intracellular alkalization induced by NHE1 activation may enhance SOCE in HL-1 cells.

We further utilized ammonium chloride (NH_4_Cl) to evaluate the impact of intracellular alkalization on HL-1 cells. As reported previously (18, 44), the addition of 40 mM NH_4_Cl for 24 h increased the pH_i_ from 7.2 to 7.6 (Fig. 4*E*). Given that NH_4_Cl treatment for 48 h elevated pH_i_ (45), we examined the effect of 24 h-treatments with 40 mM NH_4_Cl on SOCE responses. As expected, compared to the blank control group, NH_4_Cl treatment significantly increased SOCE compared to the control group (Black *versus* red, Fig. 4*F*). Moreover, this NH_4_Cl-induced SOCE enhancement was diminished by co-administration with the SOCE inhibitor celastrol (46).

Similar to what was observed with Ang II-induced alkalization, results showed that NH_4_Cl treatment also triggered hypertrophic responses in HL-1 cells, as evidenced by elevated ANF mRNA levels and increased surface area compared to the blank control group (Fig. 4, *G* and *H*). Of note, these hypertrophic changes were markedly reduced by celastrol, suggesting that elevated SOCE under alkaline pH_i_ conditions contributes to the process of cardiac hypertrophy.

Collectively, we demonstrated that Ang II or NH_4_Cl induces intracellular alkalization, leading to augmented STIM1 activation and enhanced SOCE, resulting in hypertrophic responses in HL-1 cells. Our results thus revealed a previously less-recognized mechanism underlying the enhanced SOCE essential for NHE1-related cardiac hypertrophy (36, 37).

## Conclusions

STIM1 proteins are ubiquitously expressed multimodal stress sensors, with well-characterized mechanisms for its Ca^2+^ binding and thermal sensing. Here we unveil a new facet for the multimodal STIM1 sensor: STIM1 acts as a *bona fide* H^+^ sensor, exhibiting an apparent *pKa* value around 7.2. Independent of its Ca^2+^ binding, the extent of protonation at STIM1 H355-H407 define its activation status. Intracellular alkalization, or deprotonation at H355-H407, can directly activate the molecule, while acidic conditions render it less active, potentially impairing STIM1-dependent functions in cancer cells characterized by a lower pH. Structural dynamics of H355-H407 in response to pH variations remain unclear due to limited structural data (47), necessitating further investigation. Additionally, STIM1-HN mutations are predicted to possess high pathogenicity (48), and the potential implications of diseases associated with the STIM1-2HN mutation warrant future exploration. We further demonstrated that STIM1 may respond to intracellular alkalization by generating enhanced SOCE, leading to hypertrophic responses. This highlights the crucial role of STIM1 in coordinating H^+^ and Ca^2+^ sensing to generate appropriate SOCE responses for cellular function. These findings hold significant implications for developing novel strategies to treat diseases related to H^+^ and Ca^2+^ dysregulation, such as hypertrophy.

## Experimental procedures

### Chemicals and plasmids

The coding sequence (CDS) of STIM1_1-CC1_ and TOLLES (33) were PCR amplified from wild-type STIM1, and SRAI respectively. The pCDNA3.1 vector was linearized with restriction enzymes *BamHI* and *EcoRI*. Subsequently, these sequences were assembled to generate pCDNA3.1 STIM1_1-CC1_-TOLLES. Similarly, STIM1_1-261_-TOLLES, mScarlet-SOAR1L, mScarlet-SOAR1, mScarlet-SOAR2L, and pHmScarlet-mTurquoise2 were generated using the same method. The ER-localized super-ecliptic pHluorin (SEP) was constructed by separately adding the calreticulin signal sequence and the ER retention signal sequence KDEL (49) to the N- and C-terminus of SEP (50). To generate the relevant, STIM mutations, PCR fragments containing the desired mutations, as specified in Table 1, were first amplified using primers containing mutations from the STIM1-WT template. Subsequently, these fragments were reassembled into a pCDNA3.1(+) expression vector. All sequences were reassembled using the Ready-to-Use Seamless Cloning Kit (Sangon Biotech (Shanghai) Co, Ltd). pHmScarlet (43) and SEP (50) and were kindly provided by Professor Xu Pingyong as gifts. All constructs underwent confirmation by sequencing.

**Table 1:** Sequences of primers

Target	Forward (5′ to 3′)
F-STIM1	GGTACCGAGCTCGGATCCGCCACCATGGATGTATGCGTCCGTCTTGCCCT
R-STIM1_1-CC1_	GGTTTAATCACAGACACCATCTCATTCTCAGTACCCTCCC
R-STIM1_1-261_	GGTTTAATCACAGACACCATAAGGTCATGCAGACTCTGCT
F-SORA1L	TCCGGACTCAGATCTCGAGCTCCAGAGGCCCTTCAGAAGT
R-SOAR1L	TGGATATCTGCAGAATTCCTGCATGGACAAGG
F-pet28a-STIM1	GAAGGAGATATACCATGCATCATCATCATCATCATATGAGTGAGGATGAGAAACTC
R-EF-SAM	GTTGTCCTCTTCGCCCTTGCTCACCCCAAAGAGCACTGTATCCAGA
F-mNG	TCTGGATACAGTGCTCTTTGGGGTGAGCAAGGGCGAAGAGGACAAC
R-mNG	TAGCAGCCGGATCCTCGAGTTACTTGTACAGCTCGTCCATGCCCATCACG
F-mScarlet	GCTCGGATCCCGCCACCATGGEGAGCAAGGGCGAGGCAGTG
R-mScarlet	AGCTCGAGATCTGAGTCCGGACTTGTACAGTCCGTCCATGCC
F-TOLLES	GGGAGGGTACTGAGAATGAGATGGTGTCTGTGATTAAACC
R-TOLLES	GGATATCTGCAGAATTCTTATTTAGCTTGAGAAGGCAGC
F-Calreticulin signal	CCGGAGCTCGGATCCGCCACCATGGGATGGAGCTGTATCATCCTCTT
F-SEP	TCCCAGGTCCAACTGCAGGGATCCAGTAAAGGAGAAGAAC
R-KDEL-SEP	TGCTGGATATCTGCAGAATTCTTACAGCTCGTCCTTTTTGTAT
F-H240N	CGTTACTCCAAGGAGAACATGAAGAAGATG
R-H240N	CATCTTCTTCATGTTCTCCTTGGAGTAACG
F-H355N	AGAAGTGGCTGCAGCTGACAAATGAGGTGGAGGTGCAATATTA
R-H355N	TAATATTGCACCTCCACCTCATTTGTCAGCTGCAGCCACTTCT
F-H407N	CTGGATGATGTAGATAATAAAATTCTAACAGCT
R-H407N	AGCTGTTAGAATTTTATTATCTACATCATCCAG
F-H395-398N	CTCTTTGGCACCTTCAACGTGGCCAACAGCTCTTCCCTGGAT
R-H395-398N	ATCCAGGGAAGAGCTGTTGGCCACGTTGAAGGTGCCAAAGAG
F-H355E	AGAAGTGGCTGCAGCTGACAGAAGAGGTGGAGGTGCAATATTA
R-H355E	TAATATTGCACCTCCACCTCTTCTGTCAGCTGCAGCCACTTCT
F-H355R	AGAAGTGGCTGCAGCTGACACGAGAGGTGGAGGTGCAATATTA
F-H355R	TAATATTGCACCTCCACCTCTCGTGTCAGCTGCAGCCACTTCT
F-pHmScarlet	CCGAGCTCGGATCCGCCACCATGGTGAGCAAGGGAGAGGC
R-pHmScarlet	TCCTCGCCCTTGCTCACCATTTTATACAGCTCATCCATGC
F-mTurquoise2	GCATGGATGAGCTGTATAAAATGGTGAGCAAGGGCGAGGA
R-mTurquoise2	CTGGACTAGTGGATCCTTACTTGTACAGCTCGTCCATGCC
F-ANF	ACTGATGAGAGGGAGGCCAT
R-ANF	CCGTGGGTTGGACAGATGAA
F-GAPDH	TGTGTCCGTCGTGGATCTGA
R-GAPDH	CCTGCTTCACCACCTTCTTGA

### Cell culture and transfection

HeLa and HEK293 cells were cultured in DMEM (HyClone) supplemented with 10% FBS (AusGeneX, Australia) and 1% P/S (Thermo Fisher Scientific). HL-1 cells were cultured in Claycomb medium (Sigma-Aldrich) with the same supplements, plus 0.1 mmol/L norepinephrine (MedChemExpress), and 2 mmol/L L-glutamine (Sigma-Aldrich) (51). All cell cultures were maintained at 37 °C in a 5% CO_2_ atmosphere.

Transfection of HeLa and HEK cells was conducted using electroporation, following established protocols (31). Cells were suspended in OPTI-MEM and transferred to a 4-mm cuvette for electroporation using the Bio-Rad Gene Pulser Xcell system (Bio-Rad, Hercules, CA, USA). HEK cells underwent a voltage step pulse, while HeLa cells were subjected to an exponential pulse. Following electroporation, cells were seeded onto round coverslips in OPTI-MEM (Thermo Fisher Scientific) for 60 min before being supplemented with DMEM for further cultivation. And then experiments were carried out 24 h after transfection.

The transfection of HL-1 cells was performed according to the manufacturer's instructions (Thermo Fisher Scientific, Waltham, MA, USA) using Lipofectamine 3000. Cells were seeded onto round coverslips 1 day prior to transfection. All experiments were conducted 24 to 48 h after transfection. Before each experimental treatment, cells were incubated overnight in serum-free and norepinephrine-free medium, followed by incubation of Ang II or other drugs for another 24 h (52).

### Fluorescence imaging

Fluorescence imaging was conducted using a ZEISS Observer Z1 microscope, equipped with an X-Cite 120-Q light source (Lumen Dynamics), a 40× oil immersion objective (NA 1.3), and an ORCA Flash 4.0 V3 Camera (Hamamatsu Photonics, Hamamatsu City, Japan). The imaging solution comprised 107 mM NaCl, 7.2 mM KCl, 1.2 mM MgCl_2_, 11.5 mM glucose, and 20 mM HEPES-NaOH (pH 7.2). All experiments were performed at room temperature (20∼25 °C).

For Ca^2+^ imaging experiments, genetically encoded Ca^2+^ indicator (GECI) R-GECO1.2 (53) GEM-GECO (54), or a chemical Ca^2+^ indicator Fura-2 was used. To load Fura-2 into HL-1 cells, cells were incubated in an imaging solution containing 2 μM Fura-2-AM (Thermo Fisher Scientific) for 60 min at room temperature in the dark prior to the experiment. Acquisition of fluorescence (F) was conducted at 2 s intervals, employing specific filter sets for each indicator: R-GECO1.2 (549 ± 12.0 nm _Ex_, 630 ± 34.5nm _Em_), GEM-GECO (377 ± 25.0 nm _Ex_ and 457 ± 25.0 nm _Em_ (F_Blue_), 542 ± 13.5 nm _Em_(F_Green_)), Fura-2 (510 ± 42 nm _Em_ and 340 ± 12.5 nm _Ex_ (F_340_), 387 ± 5.5 nm _Ex_ (F_380_)). For R-GECO1.2, changes in fluorescence (ΔF) relative to basal values (F_0_) were used to indicate Ca^2+^ signals. While fluorescence ratios, F_Blue_/F_Green_ and F_340_/F_380_, were employed for ratiometric GEM-GECO and Fura-2, respectively, to present dynamic changes in Ca^2+^ levels.

For FRET measurements, CFP & YFP and mScarlet & TOLLES were used as donor & acceptor pairs, respectively, with signals captured every 10 s. Bleed through corrected FRET (FRET_c_) was calculated as (1) where FRET_c_ represents the corrected total amount of energy transfer, F_d_/D_d_ represents measured bleed-through of CFP or TOLLES into the FRET filter, and F_a_/D_a_ represents the measured bleed-through of YFP or mScarlet through the FRET filter. Calibration was performed as described before (31).

(1) $${\text{FRET}}_{c}=F_{\text{raw}\;}-F_{d}/D_{d}\times F_{\text{don}\text{or}}-F_{a}/D_{a}\times F_{\text{accept}\text{or}}$$

For FRET experiments employing CFP & YFP, CFP served as the donor and YFP as the acceptor, utilizing F_CFP_ (438 ± 12 nm _Ex_/483 ± 16 nm _Em_), F_YFP_ (500 ± 12 nm _Ex_/542 ± 13.5 nm _Em_), and FRET_raw_ (438 ± 12 nm _Ex_/542 ± 13.5 nm _Em_) filters sets. The related parameters for the CFP & YFP FRET pair were the same as before (31). To reduce variations caused by differences in expression levels of CFP or YFP, FRET_c_ values were normalized against the fluorescence of donor and acceptor (F_CFP_ and F_YFP_) denoted as NFRET (32), as delineated in Equation (2),

(2) $$\text{NFRET}={\text{FRET}}_{c}/\sqrt{F_{\text{YFP}}\times F_{\text{CFP}}}$$

For mScarlet & TOLLES, with TOLLES serving as the donor and mScarlet as the acceptor, the raw fluorescence signals (F_TOLLES_, F_mScarlet_, and F_raw_, respectively) were collected using the following filters: TOLLES (438 ± 12 nm _Ex_/483 ± 16 nm _Em_), mScarlet (575 ± 16 nm _Ex_/702 ± 12.5 nm _Em_), and FRET _raw_ (438 ± 12 nm _Ex_/702 ± 12.5 nm _Em_). For this FRET pair, the three-channel corrected FRET and the system-dependent factor G were obtained with the partial acceptor photobleaching method as previously described (28, 55, 56). The computed parameters include F_d_/D_d_ = 0.082, F_a_/D_a_ = 0.076, and G = 0.96. Normalized FRET (FRETN) was obtained by normalizing FRET_c_ against F_TOLLES_ to avoid differences in expression level. Then, system-independent apparent FRET efficiency, E_app_, was calculated with the Equation (3):

(3) $$E_{\text{app}}=\text{FRET}N/\left(\text{FRET}N+G\right)$$

pH measurements were performed using genetically-encoded indicators, including SEP-ER, pHmScarlet, and pHmScarlet-mTurquoise2. Acquisition of fluorescence was recorded at 2 s intervals, using specific filter sets for each indicator: SEP (482 ± 9.0 nm _Ex_, 520 ± 14.0 nm _Em_), pHmScarlet (559 ± 17.0 nm _Ex_, 630 ± 34.5nm _Em_) and mTurquoise2 (438 ± 12.0 nm _Ex_, 483 ± 16.0 nm _Em_). For SEP and pHmScarlet, the fluorescence (F) relative to the minimum fluorescence (F_min_), obtained at pH of 6.0, was utilized to determine the pH levels. For pHmScarlet-mTurquoise2, after the separate acquisition of fluorescence signals, the fluorescence ratio-specifically, F_mScarlet_/F_mTurquoise2_-was employed to calculate the corresponding pH_i_ using Equation (5).

The fluorescence readings from respective regions of interest (ROI) were exported from SlideBook 6.0.23 software (Intelligent Imaging Innovations, Inc) and imported into MATLAB 2014a (The MathWork) to calculate corresponding data. The resulting data were plotted using the Prism 9 software. Representative traces from at least three independent experiments are shown as mean ± SEM.

### Calibration of pH sensors and measurements of intracellular pH

To construct the standard curve, cells were incubated in calibration solutions with the pH adjusted to different values that fell in a range of 5.0 to 9.0. The calibration solution contained 10 mM NaCl, 140 mM KCl, 1 mM MgCl_2_ 20 mM HEPES, and 10 μM nigericin (cat# HY-127019), adjusted to corresponding pH values (57). Then nonlinear fitting was done according to Equation (4) (43, 58), where F_pHmscarlet_/F_TOLLES_ is shown as R. R_max_ and R_min_ are the R values obtained at pH = 9.0, or pH = 5.0, respectively. Following the calibration, the p*K*a and the slope were determined. Subsequent to calibration, the pH_i_ was calculated from raw data using Equation (5).

(4) $$R=R_{\min}+\frac{R_{\min}-R_{\max}}{1+10^{\frac{pKa-{\text{pH}}_{i}}{\text{Slope}}}}$$

(5) $${\text{pH}}_{i}=pKa-\text{Slope}\times \log\left(-\frac{R_{\max}-R}{R_{\min}-R}\right)$$

### Confocal microscopy

Confocal imaging was undertaken using a ZEISS LSM880 confocal system equipped with 63× oil objective (NA 1.4, Zeiss) and controlled by ZEN 2.3 software. CFP, YFP, and mCherry were excited by 458-, 514- and 543-nm laser respectively, and the resulting fluorescence was collected at 463 to 500 nm, 520 to 600 nm, and 580 to 637 nm. Raw images were analyzed with Image J Fiji (NIH) (59). All experiments were repeated at least three times and representative results were shown.

### Analysis of cellular surface area

Before Ca^2+^ imaging experiments, HL-1 cells loaded with Fura-2 were imaged with SlideBook software. Fluorescence images were then exported and further analyzed with FIJI software. The Freehand Selections tool was utilized to delineate cell boundaries, and the Measure module was employed to calculate the areas of the selected cells.

### Real-time polymerase chain reaction (RT-PCR) analysis

Total RNA was extracted from cells using the RNA Easy Fast Cell Kit (Tiangen Biotech). Subsequently, first-strand cDNA synthesis was conducted with the PrimeScript RT Master Mix Kit (TAKARA), following the manufacturer's protocols. Real-time PCR analyses were conducted with the 2×RealStar Power SYBR qPCR Mix (GeneStar) on an ABI QuantStudio6 Q6 System (Thermo Fisher Scientific). The expression levels of the genes under investigation were normalized to the expression of GAPDH, a commonly used housekeeping gene, to account for variations in cDNA amounts across samples. The relative expression levels were calculated using the 2^−ΔΔCt^ method, which provides a quantitative measure of the fold changes in gene expression between samples.

## Data availability

The datasets generated during the current study are available from the corresponding author upon reasonable request.

## Supporting information

This article contains supporting information.

## Conflict of interest

The authors declare that they have no conflicts of interest with the contents of this article.

## References

[1] Lyu Y., Thai P.N., Ren L., Timofeyev V., Jian Z., Park S. (2022). Beat-to-beat dynamic regulation of intracellular pH in cardiomyocytes. iScience.

[2] Stock C. (2024). pH-regulated single cell migration. Pflugers Arch..

[3] Schreiber R. (2005). Ca2+ signaling, intracellular pH and cell volume in cell proliferation. J. Membr. Biol..

[4] Berridge M.J., Bootman M.D., Roderick H.L. (2003). Calcium signalling: dynamics, homeostasis and remodelling. Nat. Rev. Mol. Cell Biol..

[5] Vaughan-Jones R.D., Spitzer K.W., Swietach P. (2009). Intracellular pH regulation in heart. J. Mol. Cell Cardiol..

[6] Swietach P., Boedtkjer E., Pedersen S.F. (2023). How protons pave the way to aggressive cancers. Nat. Rev. Cancer.

[7] Reshkin S.J., Greco M.R., Cardone R.A. (2014). Role of pHi, and proton transporters in oncogene-driven neoplastic transformation. Philos. Trans. R. Soc. Lond. B. Biol. Sci..

[8] Schwartz L., Peres S., Jolicoeur M., da Veiga Moreira J. (2020). Cancer and Alzheimer's disease: intracellular pH scales the metabolic disorders. Biogerontology.

[9] Glitsch M. (2011). Protons and Ca2+: ionic allies in tumor progression?. Physiology (Bethesda).

[10] Emrich S.M., Yoast R.E., Trebak M. (2022). Physiological functions of CRAC channels. Annu. Rev. Physiol..

[11] Prakriya M., Lewis R.S. (2015). Store-operated calcium channels. Physiol. Rev..

[12] Park C.Y., Hoover P.J., Mullins F.M., Bachhawat P., Covington E.D., Raunser S. (2009). STIM1 clusters and activates CRAC channels via direct binding of a cytosolic domain to Orai1. Cell.

[13] Yuan J.P., Zeng W., Dorwart M.R., Choi Y.J., Worley P.F., Muallem S. (2009). SOAR and the polybasic STIM1 domains gate and regulate Orai channels. Nat. Cell Biol.

[14] Hoglinger C., Grabmayr H., Maltan L., Horvath F., Krobath H., Muik M. (2021). Defects in the STIM1 SOARalpha2 domain affect multiple steps in the CRAC channel activation cascade. Cell Mol Life Sci.

[15] van Dorp S., Qiu R., Choi U.B., Wu M.M., Yen M., Kirmiz M. (2021). Conformational dynamics of auto-inhibition in the ER calcium sensor STIM1. Elife.

[16] Hogan P.G. (2024). The quest to map STIM1 activation in granular detail. Cell Calcium.

[17] Soboloff J., Madesh M., Gill D.L. (2011). Sensing cellular stress through STIM proteins. Nat. Chem. Biol..

[18] Mancarella S., Wang Y., Deng X., Landesberg G., Scalia R., Panettieri R.A. (2011). Hypoxia-induced acidosis uncouples the STIM-Orai calcium signaling complex. J. Biol. Chem..

[19] Xiao B., Coste B., Mathur J., Patapoutian A. (2011). Temperature-dependent STIM1 activation induces Ca(2)+ influx and modulates gene expression. Nat. Chem. Biol..

[20] Liu X., Wang H., Jiang Y., Zheng Q., Petrus M., Zhang M. (2019). STIM1 thermosensitivity defines the optimal preference temperature for warm sensation in mice. Cell Res..

[21] Liu X., Zheng T., Jiang Y., Wang L., Zhang Y., Liang Q., Chen Y. (2023). Molecular mechanism analysis of STIM1 thermal sensation. Cells.

[22] Hawkins B.J., Irrinki K.M., Mallilankaraman K., Lien Y.C., Wang Y., Bhanumathy C.D. (2010). S-glutathionylation activates STIM1 and alters mitochondrial homeostasis. J. Cell Biol..

[23] He X., Song S., Ayon R.J., Balisterieri A., Black S.M., Makino A. (2018). Hypoxia selectively upregulates cation channels and increases cytosolic [Ca(2+)] in pulmonary, but not coronary, arterial smooth muscle cells. Am. J. Physiol. Cell Physiol..

[24] Beck A., Fleig A., Penner R., Peinelt C. (2014). Regulation of endogenous and heterologous Ca(2)(+) release-activated Ca(2)(+) currents by pH. Cell Calcium.

[25] Tsujikawa H., Yu A.S., Xie J., Yue Z., Yang W., He Y., Yue L. (2015). Identification of key amino acid residues responsible for internal and external pH sensitivity of Orai1/STIM1 channels. Sci. Rep..

[26] Shaner N.C., Campbell R.E., Steinbach P.A., Giepmans B.N.G., Palmer A.E., Tsien R.Y. (2004). Improved monomeric red, orange and yellow fluorescent proteins derived from Discosoma sp. red fluorescent protein. Nat. Biotechnol..

[27] Choi C.H., Webb B.A., Chimenti M.S., Jacobson M.P., Barber D.L. (2013). pH sensing by FAK-His58 regulates focal adhesion remodeling. J. Cell Biol.

[28] Ma G., Wei M., He L., Liu C., Wu B., Zhang S.L. (2015). Inside-out Ca(2+) signalling prompted by STIM1 conformational switch. Nat. Commun..

[29] Shrestha N., Hye-Ryong Shim A., Maneshi M.M., See-Wai Yeung P., Yamashita M., Prakriya M. (2022). Mapping interactions between the CRAC activation domain and CC1 regulating the activity of the ER Ca(2+) sensor STIM1. J. Biol. Chem..

[30] Xie J., Ma G., Zhou L., He L., Zhang Z., Tan P. (2022). Identification of a STIM1 splicing variant that promotes glioblastoma growth. Adv. Sci. (Weinh).

[31] Zheng S., Ma G., He L., Zhang T., Li J., Yuan X. (2018). Identification of molecular determinants that govern distinct STIM2 activation dynamics. PLoS Biol..

[32] Xia Z., Liu Y. (2001). Reliable and global measurement of fluorescence resonance energy transfer using fluorescence microscopes. Biophys. J..

[33] Katayama H., Hama H., Nagasawa K., Kurokawa H., Sugiyama M., Ando R. (2020). Visualizing and modulating mitophagy for therapeutic studies of neurodegeneration. Cell.

[34] Vercoulen Y., Kondo Y., Iwig J.S., Janssen A.B., White K.A., Amini M. (2017). A Histidine pH sensor regulates activation of the Ras-specific guanine nucleotide exchange factor RasGRP1. Elife.

[35] Stathopulos P.B., Schindl R., Fahrner M., Zheng L., Gasmi-Seabrook G.M., Muik M. (2013). STIM1/Orai1 coiled-coil interplay in the regulation of store-operated calcium entry. Nat. Commun..

[36] Segin S., Berlin M., Richter C., Flockerzi R.M.V., Worley P., Freichel M., Londoño J.E.C. (2020). Cardiomyocyte-specific deletion of Orai1 reveals its protective role in angiotensin-II-induced pathological cardiac remodeling. Cells.

[37] Zheng C.B., Gao W.C., Xie M., Li Z., Ma X., Song W. (2021). Ang II promotes cardiac autophagy and hypertrophy via Orai1/STIM1. Front. Pharmacol..

[38] Paletas K., Sailer X., Rizeq L., Dimitriadi A., Koliakos G., Kaloyianni M. (2008). Angiotensin-II-dependent NHE1 activation in human monocytes. J. Am. Soc. Hypertens..

[39] Liu Y., Hitomi H., Diah S., Deguchi K., Mori H., Masaki T. (2013). Roles of Na(+)/H(+) exchanger type 1 and intracellular pH in angiotensin II-induced reactive oxygen species generation and podocyte apoptosis. J. Pharmacol. Sci..

[40] Nakamura T.Y., Iwata Y., Arai Y., Komamura K., Wakabayashi S. (2008). Activation of Na+/H+ exchanger 1 is sufficient to generate Ca2+ signals that induce cardiac hypertrophy and heart failure. Circ. Res..

[41] Yamamoto T., Shirayama T., Takahashi T., Matsubara H. (2009). Altered expression of Na+ transporters at the mRNA level in rat normal and hypertrophic myocardium. Heart Vessels.

[42] Goedhart J., von Stetten D., Noirclerc-Savoye M., Lelimousin M., Joosen L., Hink M.A. (2012). Structure-guided evolution of cyan fluorescent proteins towards a quantum yield of 93%. Nat. Commun..

[43] Liu A., Huang X., He W., Xue F., Yang Y., Liu J. (2021). pHmScarlet is a pH-sensitive red fluorescent protein to monitor exocytosis docking and fusion steps. Nat. Commun..

[44] Kazyken D., Lentz S.I., Fingar D.C. (2021). Alkaline intracellular pH (pHi) activates AMPK-mTORC2 signaling to promote cell survival during growth factor limitation. J. Biol. Chem..

[45] White K.A., Ruiz D.G., Szpiech Z.A., Strauli N.B., Hernandez R.D., Jacobson M.P., Barber D.L. (2017). Cancer-associated arginine-to-histidine mutations confer a gain in pH sensing to mutant proteins. Sci. Signal..

[46] Yuan X., Tang B., Chen Y., Zhou L., Deng J., Han L. (2023). Celastrol inhibits store operated calcium entry and suppresses psoriasis. Front. Pharmacol..

[47] Yang X., Jin H., Cai X., Li S., Shen Y. (2012). Structural and mechanistic insights into the activation of Stromal interaction molecule 1 (STIM1). Proc. Natl. Acad. Sci. U. S. A..

[48] Cheng J., Novati G., Pan J., Bycroft C., Žemgulytė A., Applebaum T. (2023). Accurate proteome-wide missense variant effect prediction with AlphaMissense. Science.

[49] Suzuki J., Kanemaru K., Ishii K., Ohkura M., Okubo Y., Iino M. (2014). Imaging intraorganellar Ca2+ at subcellular resolution using CEPIA. Nat. Commun..

[50] Sankaranarayanan S., De Angelis D., Rothman J.E., Ryan T.A. (2000). The use of pHluorins for optical measurements of presynaptic activity. Biophys. J..

[51] White S.M., Constantin P.E., Claycomb W.C. (2004). Cardiac physiology at the cellular level: use of cultured HL-1 cardiomyocytes for studies of cardiac muscle cell structure and function. Am. J. Physiol. Heart Circ. Physiol..

[52] Otaegui D., Querejeta R., Arrieta A., Lazkano A., Bidaurrazaga A., Arriandiaga J.R. (2010). Phospholipase Cbeta4 isozyme is expressed in human, rat, and murine heart left ventricles and in HL-1 cardiomyocytes. Mol. Cell Biochem..

[53] Wu J., Liu L., Matsuda T., Zhao Y., Rebane A., Drobizhev M. (2013). Improved orange and red Ca(2)+/- indicators and photophysical considerations for optogenetic applications. ACS Chem. Neurosci..

[54] Zhao Y., Araki S., Wu J., Teramoto T., Chang Y.F., Nakano M. (2011). An expanded palette of genetically encoded Ca(2)(+) indicators. Science.

[55] Zal T., Gascoigne N.R. (2004). Photobleaching-corrected FRET efficiency imaging of live cells. Biophys. J..

[56] Wang Y., Deng X., Zhou Y., Hendron E., Mancarella S., Ritchie M.F. (2009). STIM protein coupling in the activation of Orai channels. Proc. Natl. Acad. Sci. U. S. A..

[57] Demaurex N., Grinstein S., Celis J.E. (2006). Cell Biology.

[58] Ponsford A.H., Ryan T.A., Raimondi A., Cocucci E., Wycislo S.A., Fröhlich F. (2021). Live imaging of intra-lysosome pH in cell lines and primary neuronal culture using a novel genetically encoded biosensor. Autophagy.

[59] Schindelin J., Arganda-Carreras I., Frise E., Kaynig V., Longair M., Pietzsch T. (2012). Fiji: an open-source platform for biological-image analysis. Nat. Methods.

