# Supplementary material for: STIM1 functions as a proton sensor to coordinate cytosolic pH with store-operated calcium entry
Source: J Biol Chem. 2024 Oct 23;300(12):107924. doi: 10.1016/j.jbc.2024.107924 (PMC11626807; doi:10.1016/j.jbc.2024.107924)

Supplementary Figure 1. Overview of the STIM constructs and design strategies of the FRET tools for monitoring STIM1 activation *in situ* and *in cellulo*.

A) Overview of the STIM constructs used. Abbreviations: SC, STIM1$_{1\text{-}CC1}$ constructs (STIM1$_{1\text{-}310}$); SCS, short version of SC (STIM1$_{1\text{-}261}$); PM-SC, PM-localized SC constructs; SP, signal peptide; TP, target peptide; NT, N terminus; EF-SAM, EF-hand and sterile alpha motif domain; TM, transmembrane region; CC1, coiled-coil 1; SOAR, STIM-Orai–activating region, or STIM1$_{344\text{-}442}$; SOAR1L, a longer version of SOAR, or STIM1$_{343\text{–}491}$; SOAR2L, STIM2$_{436\text{–}533}$; YFP, yellow fluorescent protein. CFP, cyan fluorescent protein; TOLLES, a CFP variant named Tolerance of Lysosomal Environments.

B) A cartoon interpreting the FRET strategies used to monitor the activation of STIM1. Co-localization (top panel) and FRET signals (bottom panel) between SC and SOAR1L under basal and activated states. FP, fluorescent protein.

C) Schematic illustration of the strategies used to construct PM-SC1111 or PM-SC1112. Compared to SC, PM-SC constructs incorporate the following SP or TP to ensure effective PM localization: an extracellular TP derived from CD8A$_{1\text{-}21}$, which ensures proper orientation towards the external environment; a PM-trafficking TP from Kir2.1$_{233\text{–}252}$ and an ER-exporting TP Kir2.1$_{374\text{-}380}$ that both facilitate the trafficking of STIM constructs from the ER to the PM. Of note, the SPs of both SC and PM-SC constructs are cleaved by the cell after the proteins mature and are properly localized.

Supplementary Figure 2. Characterization and application of acid-resistant or pH-sensitive fluorescence probes in HeLa cells.

A) Typical traces showing pH responses in the ER or cytosol, indicated by co-expressed pHluorin-ER (green) and pHmScarlet (red), respectively. pHluorin-ER is an ER-targeting version of a cytosolic pH sensor (n = 3, error bars denote SEM).

B) pH titrations of pHmScarlet or pHmScarlet-mTurquoise2. Left: typical traces; Right: dose response curves (n = 3).

C) In cells co-expressing TOLLES and mScarlet, representative traces demonstrating that changes in pH_i minimally influence the fluorescence of TOLLES and mScarlet (left), and basal FRET signals between these proteins are minimal (right) (n = 3, error bars denote SEM).

D) Typical confocal images depicting the ER-like distribution of transiently expressed STIM11-CC1-TOLLES in HEK cells. Scale bar, 10 μm (n = 3).

Supplementary Figure 3. Alignments of the CC1 and SOARL region of STIM1 and STIM2, respectively

Red highlights, identical histidine residues; cyan highlights, non-conservatively differing residues; and grey highlights, conservatively differing residues.

Supplementary Figure 4. pH and Ca^{2+} responses of STIM1 variants in HeLa SK cells.

A) The FRET response between mScarlet-SOAR1L and SC-TOLLES (black) or the shorter SCS-TOLLES (red) showed no significant difference. Left: Representative traces; Middle and right: statistics (n = 3, ns, $P > 0.05$, unpaired Student's *t*-test, error bars denote SEM).

B) STIM1-H395N-H398N mutation had no effect on *in situ* FRET responses between SCS-TOLLES and mScarlet-SOAR1L. Left: Typical traces; Middle and right: statistics (n = 3, ns, $P > 0.05$, unpaired Student's *t*-test, error bars denote SEM).

C) Typical confocal images depicting STIM1-2HN subcellular localization before and after ER Ca^{2+} store depletion. ER Ca^{2+} store was induced by a 5-minute bath application of 2.5 μM IONO

(Scale bar: 10 μm, n = 3).

Supplementary Figure 1

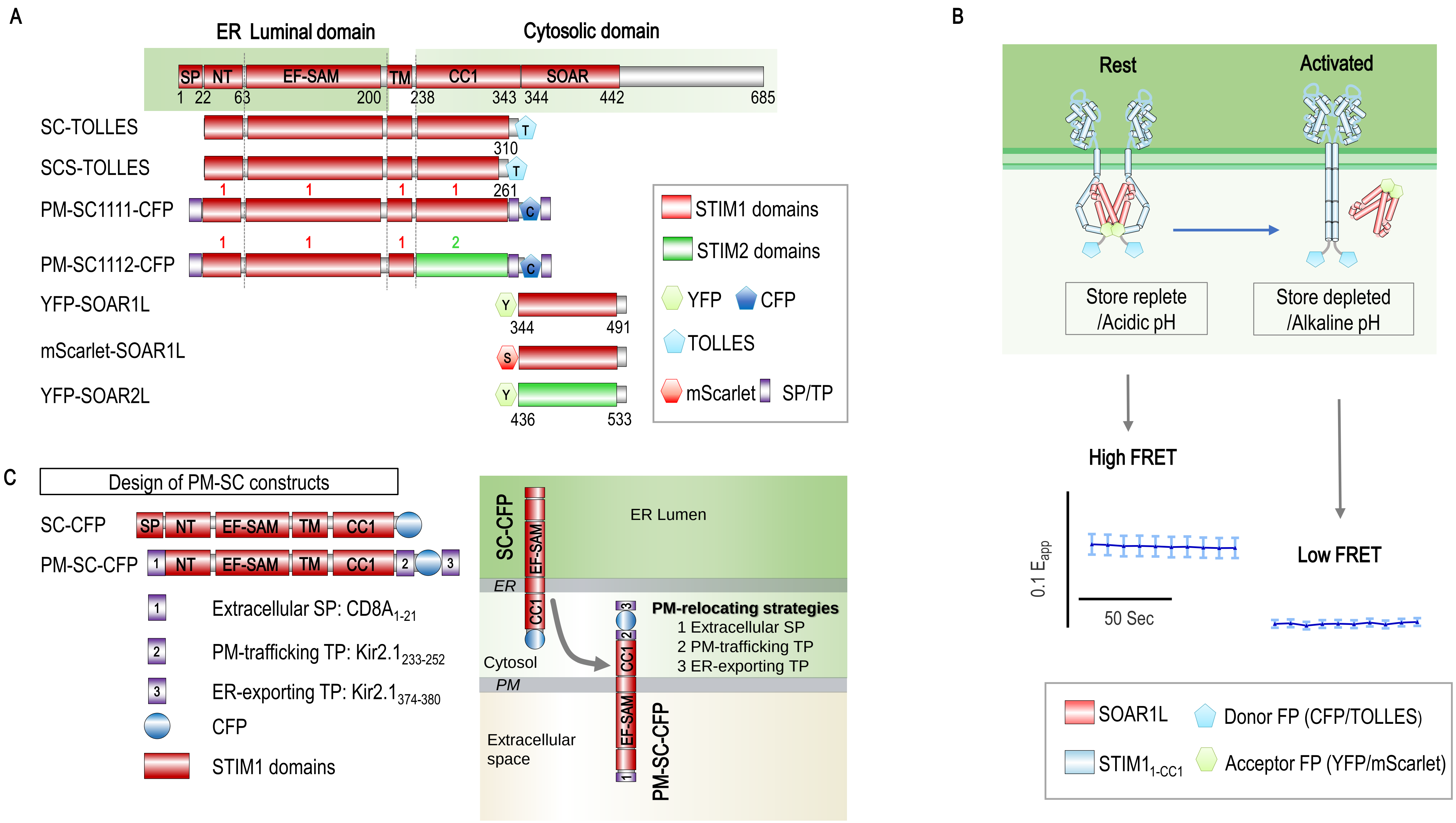

Supplementary Figure 2

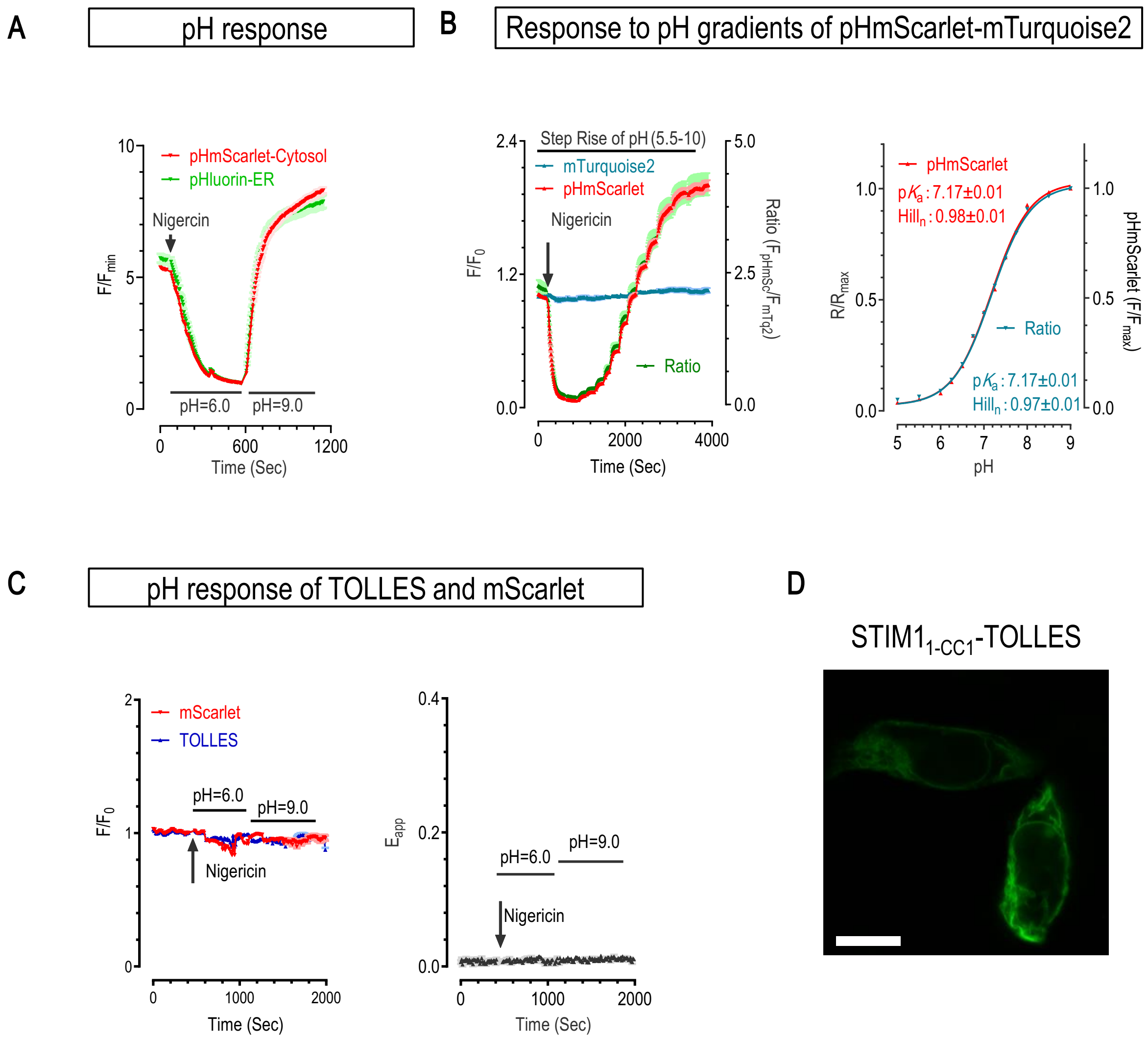

Supplementary Figure 3

A

CC1:

STIM1$_{233-310}$ QNRYSKEHMKKMMKDLEGLHRAEQSLHDLQERLHKAQEEHRTVEVEKVHLEKKLRDEINLAKQEAQRLKELREGTENE

STIM2$_{324-352}$ QNKTSKEHVAKMMKDLESLQTAEQSLMDLQERLEKAQEENRNVAVEKQNLERKMMDEINYAKEEACRLRELREGAECE

SOARL:

STIM1$_{343-420}$ APEALQKWLQLTHEVEVQYYNIKKQNAEKQLLVAKEGAEKIKKKRNTLFGTFHVAHSSSLDDVDHKILTAKQALSEVT

STIM2$_{434-511}$ VPDALQKWLQLTHEVEVQYYNIKRQNAEMQLAIAKDEAEKIKKKRSTVFGTLHVAHSSSLDEVDHKILEAKKALSELT

STIM1$_{421-491}$ AALRERLHRWQQIEILCGFQIVNNPGIHSLVAALNIDPSWMGSTRPNPAHFIMTDDVDDMDEEIVSPLSMQ

STIM2$_{512-581}$ TCLRERLFRWQQIEKICGFQIAHNSGLPSLTSSLYSDHSWVVMPRVSIPPYPIAGGVDDLDED-TPPIVSQ

Identical histidine | Conservatively differing amino acids | Non-conservatively differing amino acids

Supplementary Figure 4

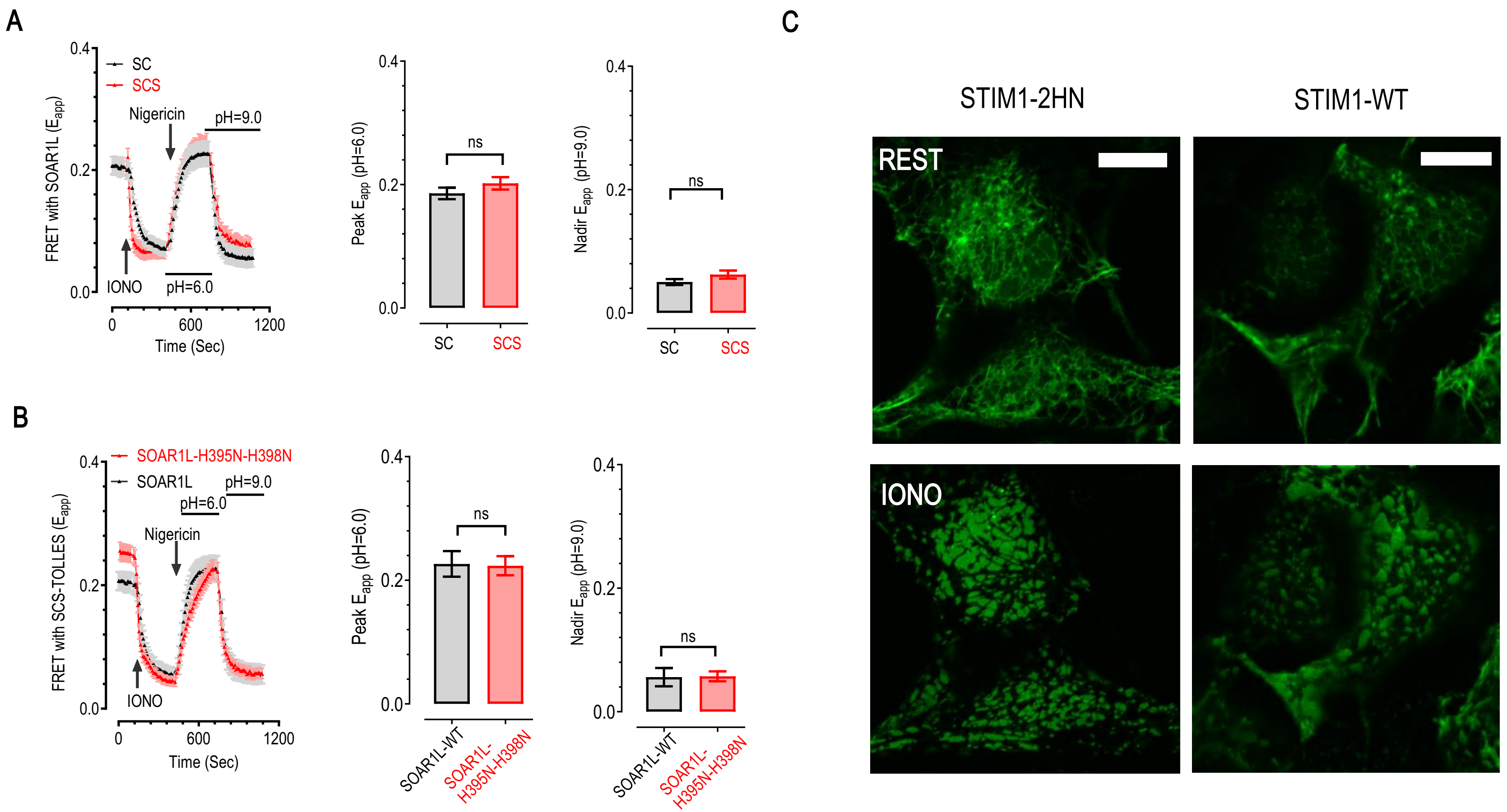

Supplement: Supplemental Figs. S1–S4 [file mmc1.pdf]
